# Increased flooded area and exposure in the White Volta river basin in Western Africa, identified from multi-source remote sensing data

**DOI:** 10.1038/s41598-022-07720-4

**Published:** 2022-03-08

**Authors:** Chengxiu Li, Jadunandan Dash, Moses Asamoah, Justin Sheffield, Mawuli Dzodzomenyo, Solomon Hailu Gebrechorkos, Daniela Anghileri, Jim Wright

**Affiliations:** 1 School of Geography and Environmental Science, University of Southampton, Southampton, UK; 2 School of Public Health, University of Ghana, Accra, Ghana

**Keywords:** Natural hazards, Environmental impact, Hydrology

## Abstract

Accurate information on flood extent and exposure is critical for disaster management in data-scarce, vulnerable regions, such as Sub-Saharan Africa (SSA). However, uncertainties in flood extent affect flood exposure estimates. This study developed a framework to examine the spatiotemporal pattern of floods and to assess flood exposure through utilization of satellite images, ground-based participatory mapping of flood extent, and socio-economic data. Drawing on a case study in the White Volta basin in Western Africa, our results showed that synergetic use of multi-temporal radar and optical satellite data improved flood mapping accuracy (77% overall agreement compared with participatory mapping outputs), in comparison with existing global flood datasets (43% overall agreement for the moderate-resolution imaging spectroradiometer (MODIS) Near Real-Time (NRT) Global Flood Product). Increases in flood extent were observed according to our classified product, as well as two existing global flood products. Similarly, increased flood exposure was also observed, however its estimation remains highly uncertain and sensitive to the input dataset used. Population exposure varied greatly depending on the population dataset used, while the greatest farmland and infrastructure exposure was estimated using a composite flood map derived from three products, with lower exposure estimated from each flood product individually. The study shows that there is considerable scope to develop an accurate flood mapping system in SSA and thereby improve flood exposure assessment and develop mitigation and intervention plans.

## Introduction

Flood events occur frequently in many regions of SSA due to high climate variability and associated extreme precipitation events^1^. Growing population along with continued socio-economic changes such as urbanisation and farming expansion increase exposure to flooding^2–5^ and result in large increases in flood risk^6–8^. Flooding poses long-term challenges to livelihoods in SSA, not only through loss of lives, destruction of farmlands and infrastructure^6^, but also disease outbreaks and worsened food and water security^1^.

The damaging impacts on livelihoods can be reduced by proper flooding mitigation strategies that are guided by sufficient flood hazard assessment, monitoring, and early warning. However, lack of monitoring and information on flood extent and flood exposure hampers spatial targeting of effective mitigation strategies. Flood exposure, defined as population and assets located in flood-prone areas^9^, however has received little attention to date (Smith et al. 2019^10^). The few flood exposure assessment studies either mainly assess broad land cover classes such as 'urban area'^11^ or only use population map layers^10^, whilst very few focus on infrastructures such as roads and water supplies. A comprehensive flood exposure assessment is fundamental for flood risk assessment and key for developing flooding mitigation strategies, but also particularly crucial in data-scarce SSA, the only global region showing increasing flood mortality rates since 1990^12^. Accurate flood mapping and flood exposure assessment can help address this increase in mortality rates^13^.

The availability of accurate historical and current information on flood hazard events is particularly limited in SSA^14^. Flood hazard models have been implemented for flood forecasting and monitoring^15,16^. However, the accuracy of model-based flood hazard maps is restricted as it depends on the accuracy of various input data such as meteorological and topography data, thereby leading to greater uncertainty from error propagation^11^. In addition, these flood hazard models tend to focus on the national or regional scale that is not designed for local-scale estimation where the impacts are experienced and local-level decisions are required^11^. Global-scale flood datasets derived from satellite data are available, such as the MODIS NRT Global Flood Product^17^, the MODIS Global Flood Database^18^, and the Global Flood Detection System^19^. These datasets again have coarse spatial resolution and validation of them is highly challenging, particularly in data-sparse regions such as SSA^14^ where accurate data on flood extent at the local scale is greatly needed for risk management^20,21^.

Progress has been made to monitor flood inundation at the local scale and over the long term, particularly with the development of Earth Observation (EO) systems with increased revisit frequency and higher spatial resolution that are increasingly used in operational disaster monitoring systems^22^. Synthetic Aperture Radar (SAR) is particularly useful for flood mapping since it can provide frequent observations^23,24^ thanks to its capability to monitor land in almost any weather conditions^25,26^. Flooded areas generate a low backscatter signal and appear to be dark in SAR images, which makes them distinguishable from other land cover classes such as agricultural land or built-up areas. Many studies have used SAR images to map flood inundation^27^, using algorithms such as histogram thresholding or clustering^28^, change detection^29,30^, and time series analysis^26^. To successfully map inundation areas using such algorithms, defining a robust and objective threshold is critical as results can be sensitive to the thresholds used^31^. Currently, most studies are either based on a universal threshold value that may be unsuitable for specific sub-regions, or dependent on user-defined empirical analysis, which makes it difficult to apply in different study regions. Our study, however, proposes an approach that defines a threshold in an objective way, that is, use optical satellite images to define thresholds for SAR-based flood mapping techniques (Sect. 3.1.1). Optical images can detect water bodies^32–34^, but tend to underestimate flooded areas as they are subject to cloud cover during the rainy and flood season^20^. Spatial overlap of water bodies detected from optical images with SAR images can demonstrate the range of backscatter signal in SAR images, and can therefore be used to define thresholds objectively that are tailored for local scale application (Sect. 3.1.1). Alongside using a combination of optical images and SAR images to improve existing algorithms for flood mapping using SAR data, our study also derived flood extent and calculated total inundation area from both optical and SAR images, which provide benefits of long-term coverage of optical imagery while addressing cloud cover issues via the use of SAR.

In addition, the lack of local ground data that can be used to evaluate the accuracy and limitations of satellite data-derived flood extent has been another major drawback in previous studies. Most studies assess accuracy via inter-comparison of satellite-based flooding maps^20,22^, for example, by using optical images to evaluate the accuracy of flood mapping from radar images^20^. However, this approach carries limitations as optical imagery may be affected by high cloud occurrence. Alongside using high-resolution satellite data for assessing accuracy, this study also collected ground data through participatory mapping to assess the accuracy of satellite-based flood maps. Participatory mapping, which engages local knowledge and expertise and allows local communities to delineate flood-affected extent on provided basemaps, has been widely recognized as an effective tool to collect and understand flood extent on the ground^35^. Involving communities’ knowledge of floods through participatory mapping is critical in the data-scarce SSA context as communities experience flooding first hand. Despite the value of local experience and knowledge, very few attempts have been made to combine flood extent derived from satellite data and through participatory mapping^1,36,37^ for evaluating satellite-derived flood extent accuracy. Such a comparison can not only enable accuracy assessment of satellite-derived flood extent, but also indicate flood-prone areas associated with high impacts for local communities.

This research, for the first time, employed a combination of top-down approaches based on multi-source satellite images and a consultative approach via participatory mapping to map flooded areas and their dynamics. Further, it investigates the scale and severity of population, infrastructure and farmland exposure to flooding in the White Volta basin in Ghana. Specifically, this study aims to:

- Map flood area at fine spatial scale (i.e., 10 m) using multi-source satellite images (including Sentinel-1, Sentinel-2 and Landsat-8) and analyse flood area dynamics through comparison with existing global flood datasets (e.g., MODIS NRT Global Flood Product, The European Commission’s Joint Research Centre (JRC) Global Flood Database, and JRC Global Surface Water dataset) over 2000–2020.
- Evaluate the accuracy of satellite-derived flood extent (from this study and the existing global flood datasets), through comparison with participatory mapping outputs.
- Estimate flood exposure by combining the satellite-derived flood datasets with socio-economic data including high-resolution population density (100 m and 30 m), land use (30 m) and key infrastructures.

## Study design and study area

**Figure 1:**
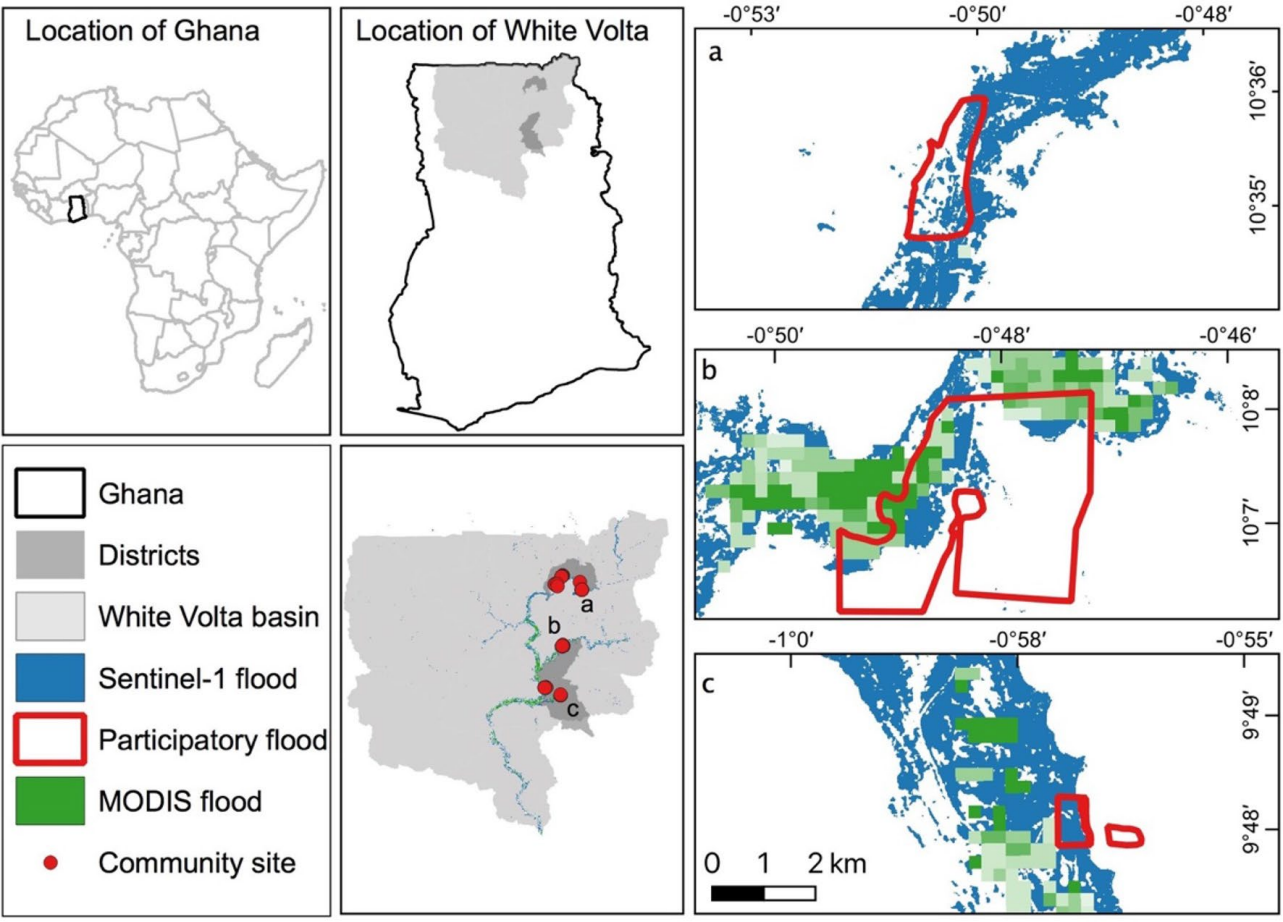
Location of the study area and maximum flood extent in September 2020 retrieved using Sentinel-1 data (blue) and MODIS NRT flood dataset (green) and identified by local participants (red boundary) using participatory mapping. The map was made with QGIS.

This study is a component of a wider program that employed a mixed-methods monitoring flooding events and examining its impacts on livelihoods, healthcare utilisation, and water point quality^38^ in White Volta catchment in Western Africa. The study area White Volta catchment is located in Ghana (Fig. 1), a subbasin of the Volta river basin with most of the population relying on rain-fed agriculture^39^. The region is prone to floods from torrential rainfall, potentially coupled with scheduled overspill from the Bagre Dam, Burkina Faso^40^ with devastating effects on the livelihoods of the poor communities living in the basin. Flood risk affects particularly the North-eastern, Northern and Savannah regions of the White Volta Basin^39^. Floods along the main course of the White Volta river have been a recurrent annual phenomenon from August to October in the recent decade^41^. Communities in northern Ghana are more vulnerable to flood hazards, with lower adaptive capacity and severer food insecurity than those in other parts of Ghana^40^. The region, however, has seldom been studied to understand flood hazards and develop adaptation measures appropriate to local conditions^40^. The current flood early warning system operated by the National Disaster Management Organization (NADMO) has not been informed by comprehensive data on flood extent at a high spatial resolution^41^.

## Data and methods

### Mapping the spatial and temporal distribution of flooding

We firstly mapped the monthly maximum flood extent at a fine spatial scale (10 m) during the wet season (July–September) using Sentinel-1 radar data available from 2016 to 2020 and optical satellite data from Sentinel-2 and Landsat-8. Secondly, we assessed the spatiotemporal flood dynamics by combining outputs from this study with existing flood datasets (the JRC Global Surface Water dataset, MODIS NRT Global Flood product, and MODIS Global Flood Database). Thirdly, we used the high-resolution PlanetScope dataset and collected ground data through participatory mapping to evaluate the accuracy of flood extent from the satellite data, and finally assess flood exposure through integration with land use, population and infrastructure map layers.

#### Flood mapping using Sentinel-1 data, Sentinel-2 and Landsat-8 data

We used the Sentinel-1 Synthetic Aperture Radar (SAR) C-band (5.4 GHz) data provided by the European Space Agency (ESA)^42^. This global dataset has a 12 or 6 day revisit cycle depending on the availability of Sentinel-1B imagery^43^ with a spatial resolution of 10 m. The level-1 Ground Range Detected (GRD) pre-processed product in Google Earth Engine was accessed and processed for the White Volta Basin, comprising 2148 scenes from 2016–2020. Only descending pathways were available in the study area and VV polarized data were used because of higher accuracy in detecting floods compared to VH, based on previous studies^20,44,45^.

We examined the combination of three algorithms to map flooded areas, namely image thresholding method^46,47^, the Change Detection and Thresholding (CDAT)^44,48^ and the Normalized Difference Flood Index (NDFI) algorithms^26^. The flooded areas identified consistently from both the CDAT and NDFI detection algorithm was found to achieve higher flood mapping accuracy than alone^20^. The CDAT algorithm uses multi-temporal SAR imagery and classifies flooded areas based on difference in backscatter between flood/post-flood and reference non-flooded images^44,48^. The NDFI also implements change detection principles, but combines a time series of images to compensate for changes in land cover and soil moisture, defined as in Eq. (1) below^26^. The image thresholding method was also examined in this study, as it is a straightforward approach based on a single image and setting pixels as flooded when the backscatter coefficient is lower than a certain threshold value^46,47^.

$$\mathrm{Equation }\,\left(1\right):\mathrm{ NDFI}= \frac{\mathrm{mean}\, \sigma 0\left(\text{"reference"}\right)-\mathrm{min}\, \sigma 0 ("\mathrm{reference}+\mathrm{flood}" )}{\mathrm{mean}\, \sigma 0\left(\text{"reference"}\right)+\mathrm{min}\, \sigma 0 ("\mathrm{reference}+\mathrm{flood}" )]}$$

Selecting non-flood reference images is key for change detection techniques^49^. We selected images in June given that this is a dry month with no flooding recorded in this area during 2016–2020 according to visual inspection of Sentinel-1 images. In addition, vegetation phenology in June is close to that in the flooding season starting from July, which minimizes the impact of seasonal variation in vegetation on backscattering. Mean value composites were generated for the reference month of June and a monthly minimal value composite image was generated for post-flood scenes to capture the maximum flood extent area.

Defining threshold values is another critical step for accurately mapping flood extent using the above three methods. For the change detection and NDFI methods, threshold values are normally determined by analysing a histogram of the distribution of backscatter difference or the NDFI index. The threshold criterion is defined based on various empirical analyses^26,44^. Normally, the threshold criterion is used to classify flooding when the values are less than the mean pixel value minus the standard deviation of the entire image, times a coefficient of 1.5^26,44^. We examined the feasibility of this commonly used threshold in our study area, as well as proposed a new approach to objectively retrieve threshold fit to local case studies. We firstly introduced sub-basin boundaries, as our study area covers a relatively large spatial domain with heterogeneous land cover types. We then calculated the thresholds described above at sub-basin level for each month. In addition, to define a more objective threshold appropriate to this study, water bodies identified via optical satellite images Sentinel-2 and Landsat-8 indices, and the JRC Global Surface Water dataset (Pekel et al. 2016^50^) were used to define the thresholds for the Sentinel-1 images. Water bodies were classified using an algorithm with three widely used indices: Normalized Difference Vegetation Index (NDVI), Enhanced Vegetation Index (EVI) and the Modified Normalized Difference Water Index (mNDWI): *Water* = *(mNDWI* > *EVI or mNDWI* > *NDVI) and EVI* < *0.1*^51^*.* We calculated the distributions of these indices for detected water bodies for each of the three flood detection methods (i.e., backscatter coefficient, backscatter coefficient difference between pre-flood and post-flood, and NDFI). For each index, the 95th percentile (5% percentile for NDFI distribution) was used as a threshold because this threshold enables the detection of 95% of water bodies derived from optical images. We examined histogram threshold criteria from previous studies^26,44^, as well as our own criterion based on optical images.

We applied a median 3 × 3 pixel filter to remove speckle effects in SAR images for a smoother image^20^. In addition, all clusters smaller than 10 pixels are excluded to reduce spurious flooded areas as the minimum mappable unit is clumps of approximately 10 pixels. Finally, any pixels falling on a slope of > 5° via NASA's Shuttle Radar Topography Mission (SRTM) elevation dataset^52^, where a flood would be unlikely, were excluded^18^. Flood mapping in urban areas, dense forest is challenging with Sentinel-1 data because of double-bounce effects, however, as the White Volta basin is dominated by savanna and crop fields that were normally submerged under floods, and flooded forest and urban area is not extensive therefore was not considered in this study.

#### Existing global flood datasets and CHIRPS precipitation data

Existing global satellite-derived flood datasets were compared with our Sentinel-1-derived flood extent data, firstly to evaluate their accuracy in flood detection in the White Volta basin, secondly to examine flood extent trends, and thirdly to generate a composite map based on the maximum flood extent from Sentinel-1 and global flood datasets. In addition, precipitation trends were analyzed to understand whether precipitation alone can explain the dynamics of changing flood extent. The global flood datasets are the JRC Global Surface Water dataset (Pekel et al. 2016^50^), and two datasets based on the moderate-resolution imaging spectroradiometer (MODIS) images: the MODIS NRT Global Flood Product^17^ and the MODIS Global Flood Database^18^. The MODIS-based datasets both used the band ratio water detection algorithms to map flood area^18^. The MODIS NRT product is available daily from 2000–2020 at 250 m spatial resolution, however only data for 2016–2020 could be accessed for the White Volta Basin. Since the MODIS Global Flood Database mainly maps large flood events documented by the Dartmouth Flood Observatory (DFO) from 2000 to 2018^18^, this dataset is available on an event basis. In this study, monthly and yearly maximum flood extent was generated using the MODIS NRT product from 2016–2020, while yearly maximum flood extent was generated using the MODIS Global Flood Database from 2000–2018. The JRC Global Surface Water dataset was generated using Landsat 5, 7, and 8 satellite imagery from 2000–2019 at a higher spatial resolution of 30 m (Pekel et al. 2016^50^). It maps the location and temporal distribution of seasonal surface water over this 20-year period at monthly level, forming a proxy for monthly flood extent. Yearly maximum flood area was also calculated based on this dataset for a longer period (2000–2020). We compared monthly and yearly maximum flood extent derived from the above flood products with the Sentinel-1 flood extent from this study, and further estimated changes in flood extent over 2000–2020. The statistical significance of flood extent trends at the basin level was assessed using the Mann–Kendall (MK) test^53^. The Climate Hazards Group InfraRed Precipitation with Station data (CHIRPS) dataset was used to examine the correlation between precipitation and flood extent. This dataset is a quasi-global rainfall dataset with a spatial resolution of 0.05° covering over 30 years, derived from satellite imagery and combined with in-situ station data^54^. Specifically, yearly daily maximum precipitation summed over the White Volta basin was calculated, and a linear regression was fitted to quantify the relationship between flood extent and precipitation.

#### Accuracy evaluation

Our flood extents delineated using Sentinel-1 data were validated through two different datasets: (1) participatory flood mapping outputs; (2) flooded and non-flooded extent classified using the PlanetScope dataset. A participatory mapping campaign for flood map accuracy assessment was conducted in September 2020 during the flood period in Northern Ghana. Eight severely flood-affected sites were selected based on consultation with the Disaster Management Organisation’s (NADMO) district office during the pre-field survey period (Fig. 1). These sites were located in the Talensi and Savelugu districts in the Upper East and Northern regions of Ghana. Basemaps, generated from high-resolution Google satellite imagery with acquisition dates between 2016 and 2019, were created for each site. Map scales varied between communities from 1:5000 to 1:20,000, following recommended practice for participatory mapping^55^. In total, 10 hardcopies of basemaps covering a total area of 19 km^2^ were used for participatory mapping, with more than one map covering some sites. In each of the eight sites, the local assembly men/women were invited to draw the flooding extent. Assembly men/women are elected politicians familiar with the local environment and flood situation through interaction with the populations they represent. They were firstly asked to identify key facilities such as hospitals and schools on the hardcopy basemaps, so that they could familiarize themselves with the map. They were then asked to delineate the extent of the maximum and the most recent flooded area they had experienced. The hardcopy basemaps were then scanned, georeferenced and digitized using QGIS^56^. All digitized maps were converted into binary maps depicting flooded and non-flooded areas. As participatory flood mapping outputs represent maximum flood extent in September 2020, we therefore compared this output with monthly maximum flood extent in September 2020 derived from Sentinel-1, MODIS and Landsat satellite data for accuracy assessment. For the participatory mapping, informed consent was sought from all human subjects, followed ethical guidelines and regulations from the Faculty of Environmental and Life Sciences, University of Southampton, UK and University of Ghana. In addition, ethical approval was obtained from the two institutions (University of Southampton, approval date: 25/07/2020, Reference 54,506.A2, University of Ghana, approval date: 04/03/2020, Reference NMIMR-IRB CPN 062/19–20).

Furthermore, PlanetScope surface reflectance data, which contain four bands (blue, green, red, near infra-red (NIR)) with a spatial resolution of 3 m, were used as reference data to evaluate the accuracy of flood extent classified by Sentinel-1. Cloud-free PlanetScope images covering the Talensi site were acquired on 25th September 2020 and compared with a contemporary Sentinel-1 scene acquired on 22nd September 2020. PlanetScope images were classified into the flooded and non-flooded areas using an algorithm with three spectral indices described above^51^*.* Stratified sampling of flooded and non-flooded locations within the participatory mapping sites and area covered by PlanetScope were conducted for accuracy assessment. A total of 2000 sample points for each validation dataset was collected for accuracy assessment, calculating the kappa coefficient, producer and user accuracy for our Sentinel-1 flood extent and global flood datasets separately. When comparing participatory maps with satellite flood extent, the participatory maps were resampled to match the spatial resolution of satellite data. Similarly, the PlanetScope image was resampled to 10 m to match the spatial resolution of Sentinel-1.

### Flood exposure estimation

Spatial overlay between flood extent and map layers of population density, land use and key infrastructures were analyzed to estimate flood exposure. Two different gridded population density maps were used in this study: the High Resolution Settlement Layer (HRSL) and the WorldPop dataset^57,58^. The HRSL dataset is based on the identification of individual buildings from high-resolution satellite imagery and distribution of population census data among these buildings to produce population density maps at a spatial resolution of 30 m for the year 2015^59^. The WorldPop dataset is based on multi-variate random forest models that disaggregate areal head counts of census data at a spatial scale of 90 m. WorldPop population layers from 2016–2020 were intersected with flood extent data for the same period, while fixed HRSL population data in 2015 were overlaid with flood maps. The spatial distribution of urban and agricultural land use was identified as follows (see Table 1). (1) The extent of land use for cities, towns, villages, farmland and others were retrieved from OpenStreetMap, a global crowdsourced database of buildings and infrastructure^60^. (2) Rural, semi-urban and urban land use areas were obtained from the Global Human Settlement Layer (GHSL), produced by JRC. (3) Cropland and urban classes for 2020 were derived from the Globeland30 global land cover dataset, which has relatively good accuracy over Africa^61,62^. Similarly, the spatial distribution of key infrastructures was also used to map flood exposure including: (1) roads, dams, and water points obtained from OpenStreetMap; (2) potential geographic occupancy of domestic water points of boreholes and water wells, produced using a maximum entropy modeling technique combining observational water point data with environmental covariates to adjust for incomplete feature coverage^63^; (3) health facilities of clinics and hospitals based on the dataset from the HealthSites initiative (https://healthsites.io/), though this dataset’s coverage might not be comprehensive. To examine the impact of using different flood datasets on exposure assessment, flood maps derived from Sentinel-1 and existing global flood datasets were intersected with population, land use, and key infrastructure map layers separately. To preserve spatial heterogeneity and give flood maps a common spatial domain, Landsat satellite data (30 m) and the MODIS flood product (250 m) were resampled to match the finest spatial resolution dataset, namely the 10 m resolution of Sentinel-1. Similarly, when estimating flood exposure, population, land use and key infrastructure layers and flood datasets were resampled to match the finest spatial resolution of any input dataset. All satellite data, population data, land use dataset were accessed and processed in the Google Earth Engine cloud-computing platform (Gorelick et al., 2017^64^) while further statistical analyses were performed in R version 4.1.2(R Core Team, 2018^65^).

**Table 1:** Socio-economic variables and datasets used for flood exposure analysis.

Socio-economic variables	Dataset	Variable retrieved		Spatial resolution	Year	Reference
Population	High-Resolution Settlement Layer (HRSL)	Population density		30 m	2015	^59^
	WorldPop	Population density		90 m	2020	^57,58^
Land use	OpenStreetMap	Urban area	Building footprints, residential, cities, etc	–	–	^60^
		Rural area	Villages, towns			
		Farm	Farm			
	Global Human Settlement Layer (GHSL)	Rural, semi-urban, urban areas		-		^66^
	Globeland30	Croplands, built-up area		30	2020	
Key infrastructures	OpenStreetMap	Roads, schools, hospitals,dams, parking, water wells, boreholes, etc		–	–	^60^
	HeathSites	Pharmacy, hospital, clinic		–	2020	https://healthsites.io/
	Domestic water points (Potential geographic occupancy)	Boreholes Water wells		1 km	2020	^63^

## Results

### Flood area delineation from multi-source satellite data

#### Flood mapping using Sentinel-1

**Figure 2:**
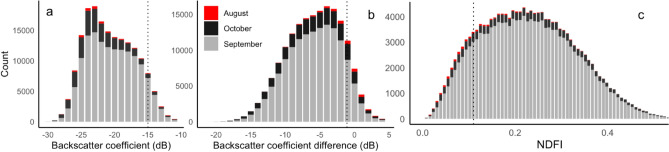
Histograms showing the distribution of backscatter coefficient (left) of water bodies, backscatter coefficient difference (middle), and NDFI (right) between pre-flood (June) and post-flood periods identified using optical satellite data (Sentinel-2 and Landsat-8) for August, September and October 2020. Dashed line indicates 95th percentile of backscatter coefficient distribution, 5% percentile of NDFI distribution.

When we implemented the NDFI and CDAT algorithms and histogram thresholds from previous studies^29,44^, we found that standalone implementation of the NDFI and CDAT algorithms gave larger flood extent estimates than combined algorithms, with a particularly high false detection rate for the NDFI algorithm (Fig. S1). Considering the high false detection rates for these existing algorithms, we therefore only present flood mapping outputs hereafter based on the thresholds defined using optical satellite images. For defining thresholds, we firstly present the frequency distribution of three indices (i.e. Sentinel-1 backscatter coefficient, backscatter coefficient difference (pre-flood and post-flood), and NDFI) within water bodies detected by optical satellite images (Fig. 2). We found that the highest pixel counts for floodwater were shown in September, followed by October and August in 2020. The backscatter coefficient of water bodies ranged from -13 to -30, and the backscatter coefficient difference between pre-flood and post-flood imagery ranged from -20 to 0.5, whilst the NDFI of water bodies ranged from 0.0625–0.5. Using 95th percentile as a threshold, a value of -15 for backscatter coefficient, -1.1 for backscatter coefficient difference and 0.14 for NDFI was identified as threshold for classifying flood extent using Sentinel-1 images (Fig. 2). When we apply these three thresholds to the image thresholding, NDFI and CDAT algorithms respectively, inconsistencies relative to topography in mapped flooded areas were reduced for all three methods (Fig. S1). Combining the image thresholding method with the CDAT and NDFI change detection algorithms particularly reduced the false detection of flooding (Fig. S1). Therefore, only areas identified as flooded by all three methods were used in subsequent flood product comparisons, accuracy assessment and flood exposure assessment.

#### Comparison with MODIS and JRC datasets

**Figure 3:**
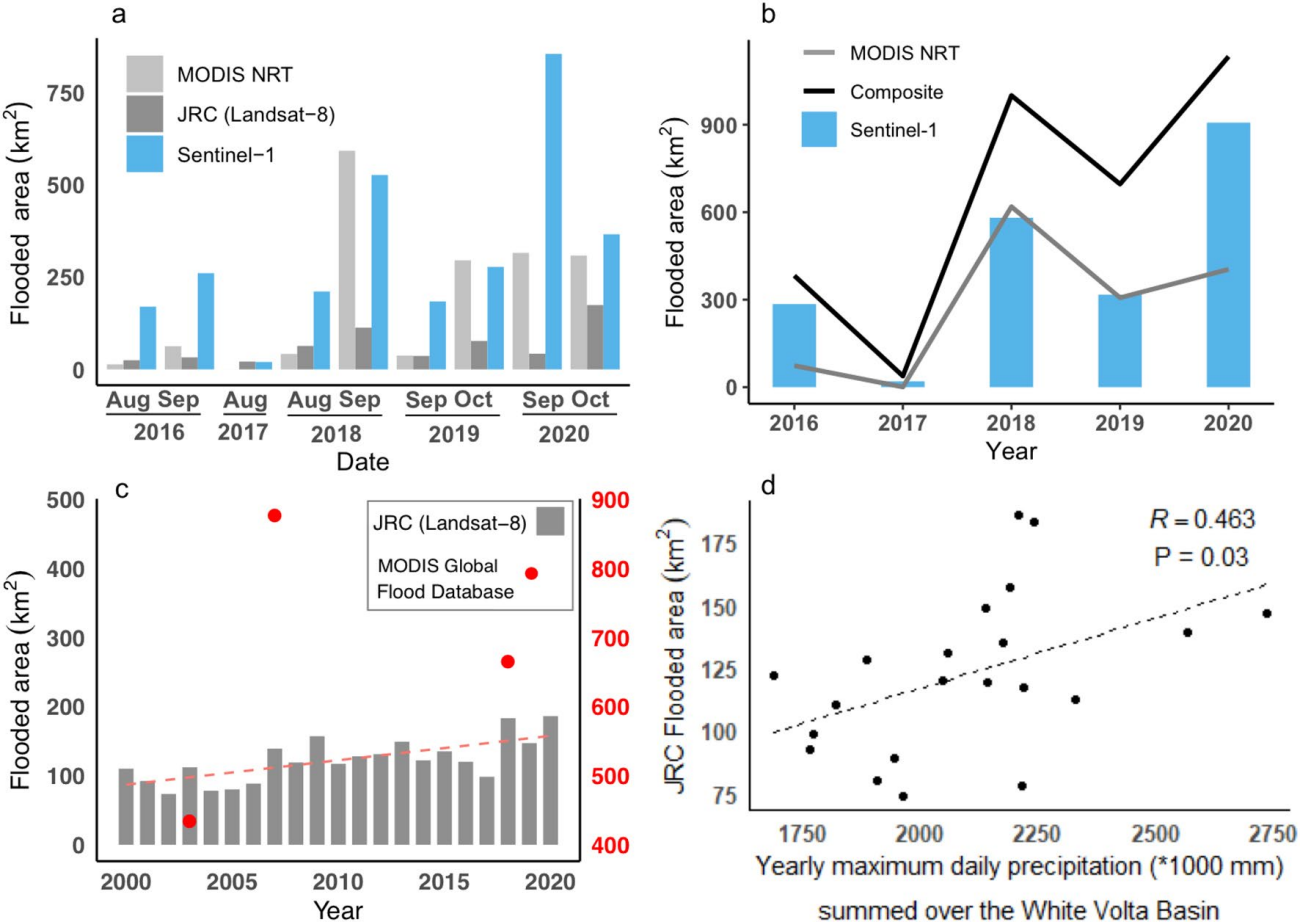
Comparison of flooded area estimated using Sentinel-1 from this study and existing global flood datasets (MODIS NRT Global Flood Dataset, MODIS Global Flood Database, JRC Global Surface Water dataset). (**a**) Maximum flood area for wet seasons from 2016–2020. (**b**) Yearly maximum flood area comparison between MODIS NRT datasets, Sentinel-1 and composite flood dataset from 2016–2020. (**c**) Flood area dynamics based on the JRC Global Surface Water dataset from 2000–2020 and MODIS Global Flood Database. (**d**) Correlation between flood area based on JRC datasets and yearly precipitation.

When we mapped flood extent during the wet season from July to October 2016–2020 using Sentinel-1 imagery (Figs. 1, 3), flooding was absent in some months. When all data products were considered, the smallest flooded area was observed in 2017 with inundation only in August, while the largest area occurred in September 2020 (Fig. 3a). The timing of the most extended flood extent within year shifted from August–September in 2016–2018 to September–October in 2019 and 2020 (Fig. 3a). Sentinel-1 data with the highest resolution produced larger flood area estimates than MODIS NRT products for all but one month (September 2018) (Fig. 3a, b). The JRC Global Surface Water dataset estimated the smallest flooded area despite its finer spatial resolution (Fig. 3a). The MODIS Global Flood Database, however, only captured flood events in three years (2003, 2007, 2018) from the past 19 years (Fig. 3c). Combining the advantages of each dataset, a composite of our Sentinel-1 based flood extent and existing global flood datasets generated the largest flood area estimate (totaling 3251 km^2^ between 2016–2020) compared to each dataset separately, but still showed similar yearly variation (Fig. 3b). The different datasets all show a temporal trend of increasing flooded area over 2016–2020 (Fig. 3b). Over the longer-term period of 2000–2020, there is an increasing trend in flooded area (with an MK test of P < 0. 01), based on the JRC Global Surface Water dataset (Fig. 3c). This trend in flood extent could be explained partly by increasing precipitation since precipitation alone explained 46.3% of variation in yearly flood extent from 2000–2020 (Fig. 3d).

#### Accuracy assessment of satellite-derived flood extent

**Figure 4:**
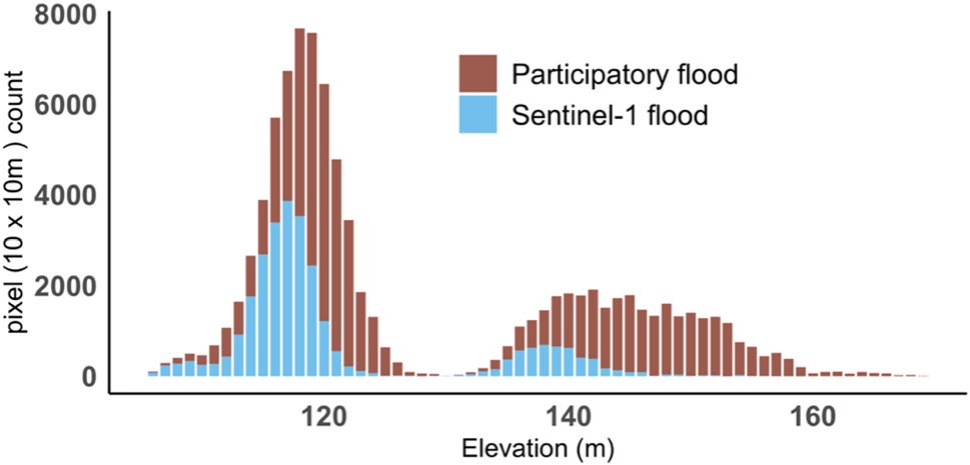
Elevation difference between flood area identified by local respondents using participatory mapping versus that identified by Sentinel-1 satellite data.

When local participants delineated the maximum and most recent flood extent, the most recent flooding extent was in September 2020 when the field campaign was taking place. This was also noted by participants to be the maximum flood extent they had experienced. Floods normally start to recede after 5–7 days according to participants. We found Sentinel-1-derived flood extent most closely matched the participatory mapping output, with an overall agreement of 0.77 (kappa = 0.55), while the agreement was lower for the MODIS NRT flooding dataset (0.43, kappa: 0.16) and lowest for the Global Surface Water data (0.3, kappa:0.008) (Table 2). Flood extent from all datasets combined shows a marginal gain in accuracy (overall agreement: 0.78, kappa = 0.57) in comparison to the participatory mapping (Table 2). When the participatory mapped flood extent was superimposed on those derived from satellite data, in all eight selected communities, local participants identified a larger flooded area extending beyond the area identified by each satellite-derived product (Fig. 2). Comparing the elevation of flooded areas identified by satellite data versus participatory mapping, the latter were on average 12 m higher than those identified by satellite data (Fig. 4). When comparing flooded extent derived from Sentinel-1 with flooded area classified using PlanetScope data, a better agreement was achieved with 92% overall agreement and a Kappa index of 0.84 (Table 3).

**Table 2:** Accuracy assessment of flood extent in September 2020 classified using Sentinel-1 imagery and via existing global flood datasets (MODIS NRT Global Flood Dataset and JRC (Landsat) Global Surface Water dataset) versus flooding delineated via participatory mapping.

	Sentinel-1	MODIS NRT	Landsat	Composite
Overall accuracy	0.77	0.43	0.3	0.78
Kappa	0.55	0.16	0.008	0.57
User accuracy	0.57	0.36	0.3	0.58
Producer accuracy	0.7	0.25	0.14	0.71

**Table 3:** Accuracy assessment of flood extent in 25th September 2020 classified using Sentinel-1 imagery versus flood extent derived from PlanetScope data in 22nd Sep 2020.

Overall accuracy	Kappa	User accuracy	Producer accuracy
0.92	0.84	0.91	0.92

The accuracy of flood extent from MODIS and Landsat images was not performed as only monthly flood extent dataset available while acquiring PlanetScope images for whole September is challenging due to high cloud cover.

### Flood exposure assessment

#### Population exposure

**Figure 5:**
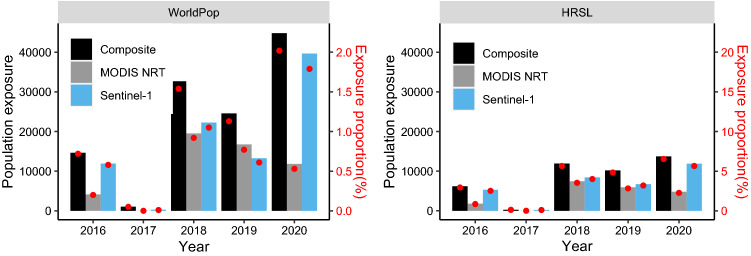
Total and proportion of population exposed to flooding from 2016–2020 estimated through spatial overlay of two population datasets (WorldPop, HRSL) with flood datasets.

We estimated that around 32,555—87, 378 people (about 4.4–15.4% of the total population) have been exposed to at least one Sentinel-1 observed event since 2016, based on the WorldPop and HRSL datasets (Fig. 5). Since population exposure increases with flood area (Fig. S3 and Fig. S4), the population exposure estimated from the MODIS NRT Global Flood Dataset was lower (19,920–52,246 people or 2.42%-9.6%) than that using Sentinel-1, whilst the greatest population exposure of around 42,272—117,717 people (5.46–20.8% of total population) was estimated using the composite flood maps (Fig. 5). Population exposure estimated using the HRSL dataset is lower than that estimated using WorldPop, however the proportion of population is higher when using HRSL data. Although the population estimates vary markedly based on the population density dataset and flood dataset, total population exposure increased over 2016–2020, with the highest population exposure in 2020 and the lowest in 2017, corresponding to the highest and lowest flood extent respectively (Fig. 5).

#### Exposure of land use types and key infrastructure

**Figure 6:**
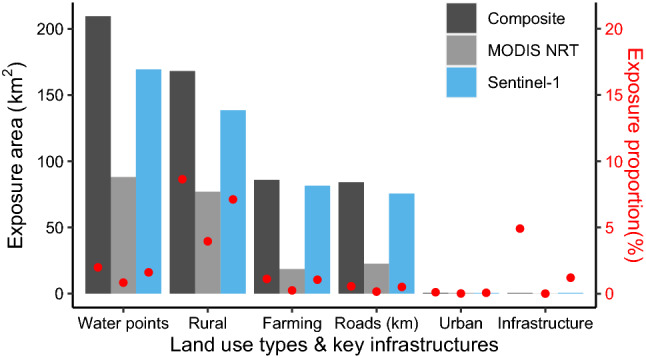
Land use areas and key infrastructures exposed to flooding in 2020.

Analysis of land use (urban, semi-urban and rural land use) and key infrastructure exposure for the largest flooding year 2020 showed that rural and farming areas are the most exposed to flooding, with 138.5 km^2^ of rural area (7.12%) and 81.42 km^2^ of farmland (1.05%). Hotspots of flood exposure of urban areas (1.2 km^2^, 0.06%) and key infrastructure such as schools and hospitals (0.06 km^2^, 1.2%) were also detected, together with roads (75.6 km, 0.5%) and potential occupancy area of water points (169 km^2^, 1.6%). These estimates are based on flood extent from the Sentinel-1 dataset, which are lower than using the composite flood map, which generates the highest flood exposure (Fig. 6). Exposure estimates were the lowest using the MODIS flood dataset: no infrastructure exposure was detected using this dataset (Fig. 6).

## Discussion

Spatially detailed mapping of flood extent and exposure assessment is generally challenging for data-poor areas such as SSA. Efficient and pragmatic methods are critical for flood mapping and helping to mitigate flood impacts, but the validation of the available data is also necessary. This study, for the first time, combines participatory mapping and multi-source satellite and socio-economic data to investigate flood extent dynamics and evaluate flood exposure in the White Volta basin in Ghana. Our results reveal both opportunities and challenges for monitoring floods using satellite data and suggest areas for further investigation. Our results further stress the need for implementing flood management strategies as we observed increasing flood hazards and exposures.

### Accuracy of flood mapping using multi-source satellite data

Flood monitoring either from optical or radar satellite data has strengths and limitations. We proposed a transferable and objective approach for flood mapping combining both radar and optical satellite images, by applying a threshold identified from optical images and a combination of algorithms including CDAT, NDFI and image thresholding of radar images. Using Sentinel-1 SAR data, estimated flood area depended on the selected threshold values, which however are very sensitive to land cover and seasonal vegetation phenological variations, leading to differences in the SAR signals for the same inundated location at varying time periods through the season and from year to year^20^. Most studies defined the threshold based on either empirical analysis or applying values from other studies’ distributional analyses^26,44^. This criterion is suitable when a large part of the study area is flooded. However, where flooding is limited, it tends to overestimate flood extent in a relatively large spatial domain with heterogeneous land cover types, such as for the White Volta basin (Fig. S1). Our study demonstrates a more objective threshold by using optical satellite data to define thresholds for SAR-based flood mapping techniques (Fig. 2). We further demonstrated that the combined use of three existing SAR-based algorithms (image thresholding, CDAT and NDFI) provides greater accuracy in flood mapping, reducing errors such as those from radar shadow in hilly terrain (Fig. S1). In addition, image thresholding could minimize the effects of land cover changes when using change detection algorithms, enabling detection of additional floods and exclusion of permanent water bodies (Fig. S1). The approach demonstrated in this study is not case-specific and therefore could be further tested and applied in other regions of the world.

Our study shows the potential of flood mapping using Sentinel-1 data, as well as the integration of multi-source satellite imagery and global flood datasets for greater accuracy in flood area estimation than when using a single optical satellite-based dataset (Table 2). Our results showed a comparable or even greater accuracy compared with existing studies^20,22^ when evaluated against satellite-based flood maps (PlanetScope images in this study). Our study further suggests that many global scale flood estimates using Landsat and MODIS images underestimate flood area and exposure (Fig. 3, Tables 2, 3). For example, the Global Surface Water dataset using Landsat images is not sufficient to assess specific flooding events as it estimates the lowest area of flooding compared with other datasets and it provides a low accuracy (0.33) in delineating flooding. The low accuracy might be related to cloud cover and coarse revisit time (16 days). However, this dataset could still provide insights into yearly variations and long-term dynamics (Fig. 1). In addition, the coarser spatial resolution of MODIS data generally captures large flood events but cannot detect small flooded areas due to its coarse spatial resolution (Fig. 1) and the MODIS Global Flood Database^18^ detected very few flood events in Northern Ghana. Our study showed that combining flood map layers from multiple sources including Sentinel-1-derived and existing flood datasets allows for more samples in time and therefore provides more complete monitoring of flood events, as a composite map showed a slight improvement in accuracy (0.78) (Fig. 4, Table 2). We therefore recommend combining existing global flood and Sentinel-1 datasets following the approach piloted in Ghana for future flood mapping, which has the potential of mapping near real-time flooding at continental or even global scale^67^.

When evaluating the accuracy of flood extent derived from this study using the Sentinel-1 dataset, we found a lower agreement between flood extent derived from satellite data and via participatory mapping than inter-comparison of satellite-based flooding maps. This low agreement indicates both strengths and limitations of satellite monitoring and participatory mapping. First of all, as a widely-used approach for environmental monitoring such as monitoring forest plantations^68^ and degradation^69^, and ecosystem services^70^, participatory mapping has been proven as an effective tool for flood risk assessment^35^ and enhancing community resilience by integrating their knowledge on the causes and mitigation strategies of flooding. In this study, local key participants tend to delineate a larger flood extent with a higher elevation than that detected via satellite imagery (Fig. 3). This indicates that Sentinel-1 data might not capture shallow inundated areas at a higher elevation, which however were identified by participants. This could further show that Sentinel-1 data might underestimate flood area, potentially because of the satellite’s revisit frequency. Even though Sentinel-1 has a revisit period of 6 days, it could still underestimate flood area, given that flooding receded within 5–7 days according to mapping participants. More frequent satellite observation is critical for flood mapping, underlining the argument above for combining flood maps from both optical and radar satellite images with different revisit patterns, so as to compensate for the limitations of each data stream and provide a denser, more complete picture of flood events. On the other hand, the participatory mapping outputs cannot be considered as a definitive measure of flood extent given the uncertainties associated with respondent perception and recall. For example, studies have shown that communities with direct flood experience tend to overestimate danger^71,72^. If such communities also over-estimate flood extent, this could account for greater flood coverage in the participatory mapping outputs compared to satellite-derived products. Indeed, we observed disagreements in drawing exact inundated boundaries between individuals during the field campaign, although final maps were created by selected key representatives best informed of their local environments and flood situations. Flooding could also have restricted participants’ movements and thus limited their knowledge of its spatial extent despite participatory mapping taking place immediately after the flood season. Participatory mapping is inherently local in scope and carries limitations for accuracy assessment across a larger region.

### Increasing flood exposure and uncertainties in its assessment

Existing global crowdsourced databases of OpenStreetMap and recently updated land cover and population datasets provide a high potential of assessing comprehensive and spatially detailed flood exposure, while also revealing the sensitivity of exposure assessment to input flood extent and socio-economic dataset used. We found that the number of people exposed to flooding increased from 2016 to 2020 irrespective of the population or flood dataset used. Increased population exposure mainly resulted from increasing flood extent (Fig. S3 and S4), so similar increases in infrastructure and land use exposure would also be expected. However, we found that flood exposure is dependent on the flood extent and population density dataset used, suggesting large uncertainties persist in flood exposure estimates, as also noted in another study^10^. For example, the large difference in population exposure is related to the way populations are mapped. WorldPop data models non-zero population density across almost the entire region, such that population is present in all flooded areas (Fig. S2). In contrast, the HRSL dataset is based on classifying building footprints from optical satellite data, thereby distributing populations across a smaller and concentrated area. Considering uncertainties persist in flood area and exposure assessment, future studies should also provide accuracy and sensitive assessment in spatial information of flood exposure estimation, which is critical for end-users looking to use emerging flood risk data sets to inform decision-making. This assessment can have implications for adopting such estimates as a scenario planning tool for decision-making. Moving forward, not only are accurate and spatially detailed datasets representing flood extent required, but also similar data sets representing people and assets. In addition, flood forecasting is critical for mitigating and managing flood risk, particularly under the projection of increased rainfall and thereby flood events in the White Volta region, as we observed that increased flood extent was closely related to greater precipitation (Fig. 3). However, predicting flooding is challenging as it involves complex processes and interactions among geomorphological, hydrological, climatic, and anthropogenic drivers^73^. Flood inundation areas derived from satellite data could be used for calibrating flood forecasting models. In addition, long-term monitoring of flooding from satellite observations could identify flood-prone areas and evaluate flood duration, which can be used for forecasting flooding using machine learning algorithms^74^.

## Conclusions

Although there are several global flood monitoring programs delivering flood locations and flooded areas near real-time, these flood products are available at a coarse resolution and their accuracy in SSA is rather limited. Since increased flood events and increased flood mortality rates have been observed in SSA, further flood extent monitoring and exposure evaluation are merited. This study developed a framework for mapping flood extent and exposure using multi-source data in the White Volta basin in Ghana. Our results showed that the combined usage of optical and radar satellite data produced better flood mapping accuracy (77% overall agreement with participatory mapping outputs) than existing global flood product (i.e., 43% overall agreement for MODIS NRT Global Flood Dataset). Our results showed that existing global flood datasets (e.g., MODIS NRT Global Flood Product and MODIS Global Flood Database), however, underestimated flood extent, indicating there is a considerable scope to develop an accurate flood mapping system in SSA. Using flood extent maps developed at fine resolution, we also demonstrated flood exposure assessment in the White Volta Basin in Ghana. We found increased population exposure and identified rural areas, farmland, and infrastructure that are prone to flooding where flood mitigation and adaptation should be prioritized. Our localized flood extent maps and flood exposure monitoring could help with flood risk assessment in SSA.

However, satellite data could underestimate inundation area depending on the revisit frequency of satellite observation and recession day of flooding. In addition, we found flooding exposure assessments are sensitive to input datasets used, such as flood extent data and population layers. Moving forward, more frequent satellite observation is critical for flood mapping. Flood maps from both optical and radar satellite images with different revisit patterns, as well as flood maps from existing flood datasets should be combined for flood monitoring as it allows for more samples in time and therefore provides more complete monitoring of flood events. In addition, flood exposure uncertainty assessment should be considered for future studies that can guide end-users for decision-making.

## Supplementary Information

Supplementary Information.

## Data Availability

Satellite imagery from Landsat-8 and Sentinel-2, Sentinel-1, the Global Surface Water dataset; The Shuttle Radar Topography Mission digital elevation data, WorldPop population dataset were accessed and processed through the Google Earth Engine platform (https://developers.google.com/earth-engine/datasets/catalog). OpenStreetMap road network data were accessed through https://download.geofabrik.de/africa.html. Catchment boundary data were accessed through the World WildLife Fund (WWF) HydroSHEDS web site https://www.hydrosheds.org/downloads. Health facilities data were accessed through HealthSite initiative https://healthsites.io/. The MODIS flood product is based on: NRT MODIS/Aqua + Terra Global Flood Product MCDWD_L3_NRT distributed from NASA LANCE. Available on-line [https://earthdata.nasa.gov/earth-observation-data/near-real-time/mcdwd-nrt]. https://doi.org/10.5067/MODIS/MCDWD-L3-NRT.061. The population dataset is accessed through www.worldpop.org. The High Resolution Settlement Layer (HRSL) is available at: http://ciesin.columbia.edu/data/hrsl/#acknowledgements [Facebook Connectivity Lab and Center for International Earth Science Information Network—CIESIN—Columbia University. 2016. Source imagery for HRSL © 2016 DigitalGlobe. Accessed 25th June 2021]. The participatory mapping output generated in this study are available on https://doi.org/10.5258/SOTON/D1956, including digitalized flooded polygon, detailed field protocol and informed consent that was sought from all human subjects.
