# Supplementary material for: Increased flooded area and exposure in the White Volta river basin in Western Africa, identified from multi-source remote sensing data
Source: Sci Rep. 2022 Mar 8;12:3701. doi: 10.1038/s41598-022-07720-4 (PMC8904518; doi:10.1038/s41598-022-07720-4)
Supplement: Supplementary file 1 — Supplementary Information. [file 41598_2022_7720_MOESM1_ESM.docx]

The Figure below shows that NDFI and CDAT algorithms alone lead to overestimate of flood extent using predefined threshold from the literatures (Cian et al. 2018; Clement et al. 2018), particularly NDFI algorithm lead to larger false detection. Applying thresholds defined by optical satellite images for NDFI and CDAT, as well as for image thresholding algorithm, the map showed that the consistent map from these three methods reduce uncertainties of flooding mapping related to hilly topography, particularly adding image threshodling method to change detection algorithms of CDAT and NDFI reduce the false detection of flooding (Compare Fig. S1b to Fig.S1c).


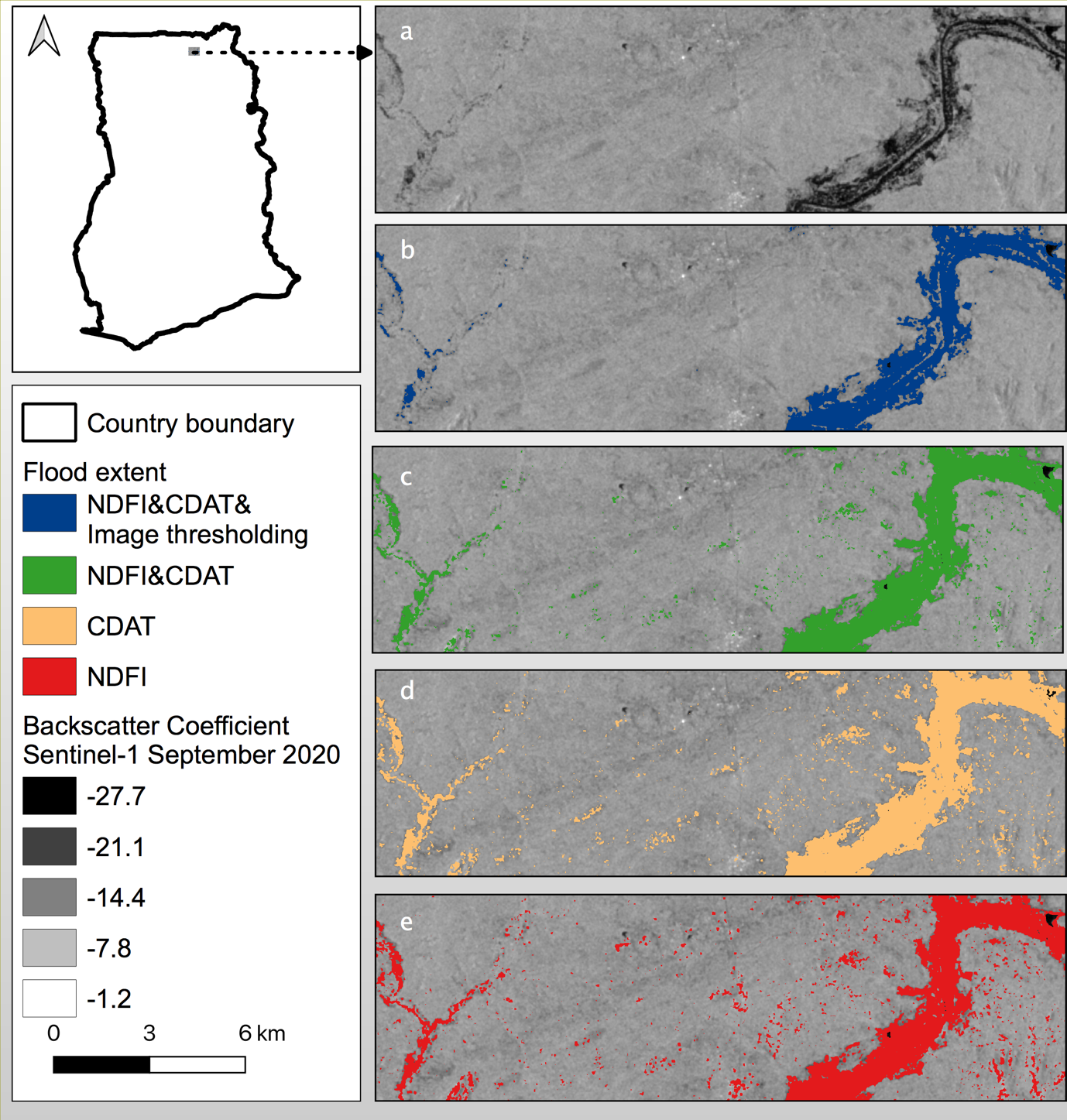


Figure. S1 Flood extent from Sentinel-1 images applying different algorithms. a) Sentinel-1 minimum backscatter coefficient composite in September 2020. b) Flood extent retrieved from approach proposed in this study, which is based on agreed extent from NDFI, CDAT and image thresholding methods using thresholds identified using optical images of Sentinel-1, Landsat-8 and JRC Global Water surface Datasets. c) Flood extent developed from the consistent extent of NDFI and CDAT algorithm using thresholds identified using optical images of Sentinel-1, Landsat-8 and JRC Global Water surface Datasets. d) Flood extent developed from only using CDAT algorithm using the threshold identified by previous studies, that is the values that are less than the mean pixel value minus the standard deviation of the entire image, times a coefficient of 1.5 (Cian et al. 2018; Clement et al. 2018) is classified as flood e) Flood extent developed from only using NDFI algorithm using the threshold identified by existing studies.

Fig. S2. Population (WorldPop) exposure to inundated area retrieved from composite map of Sentinel-1 flood extent, MODIS NRT Flood Dataset, and JRC Global Surface Water dataset in 2020.

Figure S3 Correlation population exposure calculated from HRSL dataset and total flooded area calculated from Composite, MODIS NRT and Sentinel-1 flood maps for years 2016-2020

Figure S4 Correlation population exposure calculated from WorldPop dataset and total flooded area calculated from Composite, MODIS NRT and Sentinel-1 flood maps for years 2016-2020
